# Theoretical Description of Attosecond X-ray Absorption Spectroscopy of Frenkel Exciton Dynamics

**DOI:** 10.3390/molecules28114502

**Published:** 2023-06-01

**Authors:** Tim Hansen, Tatiana Bezriadina, Daria Popova-Gorelova

**Affiliations:** 1 I. Institute for Theoretical Physics, Universität Hamburg, Notkestr. 9, 22607 Hamburg, Germany; 2 Centre for Ultrafast Imaging, Luruper Chaussee 149, 22671 Hamburg, Germany

**Keywords:** coherent electron dynamics, attosecond spectroscopy, X-ray absorption spectroscopy, energy transfer, Frenkel excitons

## Abstract

Frenkel excitons are responsible for the transport of light energy in many molecular systems. Coherent electron dynamics govern the initial stage of Frenkel-exciton transfer. Capability to follow coherent exciton dynamics in real time will help to reveal their actual contribution to the efficiency of light-harvesting. Attosecond X-ray pulses are the tool with the necessary temporal resolution to resolve pure electronic processes with atomic sensitivity. We describe how attosecond X-ray pulses can probe coherent electronic processes during Frenkel-exciton transport in molecular aggregates. We analyze time-resolved absorption cross section taking broad spectral bandwidth of an attosecond pulse into account. We demonstrate that attosecond X-ray absorption spectra can reveal delocalization degree of coherent exciton transfer dynamics.

## 1. Introduction

When a photovoltaic material or a biological complex interacts with light, a bound electron-hole pair, called exciton, can be created. Dynamics of excitons determine processes that are essential for both applications such as solar cells, light-emitting devices and field effect transistors and for the biological significant processes, such as photosynthesis [1,2]. Ultrafast energy transfer after exciton creation governs the functionality of these processes. The role of quantum coherence processes for energy transfer has been heavily debated [3,4,5,6,7,8,9,10,11]. Coherences can occur right after the exciton creation as well as be induced by the environment some time after exciton propagation [12]. Although it is not fully clear, whether coherence processes can last for hundreds of femtoseconds, they certainly play a role on femtosecond time scales before electron-phonon coupling starts to be considerable. Resolving the finest details of pure electronic processes will help to clarify their contribution to the efficiency of light harvesting.

Since recent years, it has become possible to generate attosecond X-ray pulses either at high-harmonic generation sources or at free-electron lasers [13,14,15]. Attosecond X-ray pulses offer new perspectives for revealing mechanisms of excited-state dynamics [16,17,18,19]. Sub-femtosecond temporal resolution provides an access to the ultrashort time scales of electron dynamics [20,21]. In addition, X-ray absorption spectroscopy method encodes information about a probed system with atomic selectivity and chemical sensitivity [22,23,24,25]. Although it is clear that attosecond X-ray absorption spectroscopy carries important information about dynamics in a system, the interpretation of the spectra are challenging. The bandwidths of spectral peaks are inversely proportional to the duration of a probe pulse and become several eV broad for an attosecond probe. Thus, the understanding of the information encoded by attosecond X-ray pulses relies on a thoughtful theoretical analysis. In this article, we study how attosecond X-ray absorption spectroscopy can be applied to probe coherent exciton dynamics in molecular aggregates and demonstrate that X-ray spectra provide information about exciton delocalization in such systems.

Frenkel exciton model [26] is a commonly used and robust model that successfully describes exciton dynamics in organic semiconductors, molecular aggregates or light-harvesting systems [9,27,28,29]. In such systems, interaction with light leads to an excitation of a molecule in an aggregate. Due to electron couplings between neighbouring sites, the excitation becomes delocalized over several molecules, which leads to exciton transfer through the system. Frenkel exciton transport has been studied at time scales of few hundreds of femtoseconds or longer [30,31,32,33,34]. Here, we propose to probe Frenkel exciton transport at much shorter time scales of few femtoseconds, when incoherent processes due to phonons do not start to play a role.

The article is organized as follows. In Section 2, we start with a brief introduction to coherent dynamics of Frenkel excitons. Then, we derive the general expression for time-resolved X-ray absorption cross section for Frenkel exciton system using the first-order time dependent perturbation theory. We proceed with a detailed analysis of the time-resolved X-ray absorption cross section in Section 3. The analysis relies on the study of core-excited final states that can be reached due to X-ray absorption. We connect the time-evolution of the X-ray absorption cross section to the time evolution of Frenkel excitons in Section 3.3.

## 2. Materials and Methods

### 2.1. Frenkel Exciton Dynamics

Let us consider a chain of *N* identical equidistantly aligned molecules. In the Frenkel exciton model, it is assumed that every molecule *m* can be either in the ground state $|\phi_{0}^{m}\rangle$ or in the optically-excited state $|\phi_{*}^{m}\rangle ={\hat{c}}_{m}{\hat{h}}_{m}|\phi_{0}^{m}\rangle$ [9,26,27,28,29]. ${\hat{c}}_{m}^{†}\;\left({\hat{c}}_{m}\right)$ are electron creation (annihilation) operator for site *m* and ${\hat{h}}_{m}^{†}\;\left({\hat{h}}_{m}\right)$ are hole creation (annihilation) operator for site *m*. It is assumed that the distance between the molecules is large enough to neglect exchange interaction between electrons of different molecules. The ground state of our system is

(1) $$\begin{array}{c} |\Psi_{0}\rangle =|\phi_{0}^{1}\rangle ⊗|\phi_{0}^{2}\rangle ⊗\ldots ⊗|\phi_{0}^{N}\rangle . \end{array}$$

We now introduce the basis states of Frenkel Hamiltonian

(2) $$\begin{array}{c} |\Phi_{m}\rangle =|\phi_{0}^{1}\rangle \ldots ⊗|\phi_{*}^{m}\rangle ⊗\ldots |\phi_{0}^{N}\rangle . \end{array}$$

**Figure 1:**
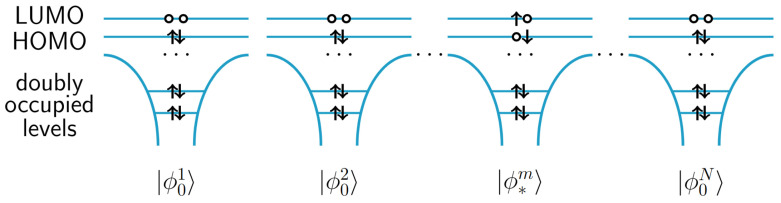
A many-body basis state $|\Phi_{m}\rangle$ of Frenkel Hamiltonian.

These are the states, in which one of molecules in the chain is excited and others are in the ground state as shown in Figure 1. The Frenkel Hamiltonian in this basis set is expressed as

(3) $$\begin{array}{c} {\hat{H}}_{0}=\sum_{m}U|\Phi_{m}\rangle \langle \Phi_{m}|+V\sum_{m}\left[\right|\Phi_{m}\rangle \langle \Phi_{m\pm 1}|+h.c.]. \end{array}$$

Here, *U* is the Coulomb energy of an electron-hole pair localized on a single site and *V* is the coupling between nearest-neighbour molecules due dipole-dipole interaction. The coupling term is responsible for the transfer of the electron-hole pair from site *m* to the sites m+1 and m−1.

The eigenstates of the Frenkel Hamiltonian, Frenkel excitons, are the states, in which the excitation is delocalized over several sites. They can be expressed as a linear combination of the basis states:

(4) $$\begin{array}{c} |\Psi_{n}\rangle =\sum_{m}c_{n}^{m}|\Phi_{m}\rangle . \end{array}$$

with corresponding eigenenergies $E_{n}$. The Frenkel exciton model assumes that only a single molecule $\tilde{m}$ gets excited due to interaction with light at time 0. The wave function right after the excitation |Ψ(t=0)〉 coincides with the basis state of the electronic Hamiltonian and is a superposition of the eigenstates $\left|\Psi \left(t=0\right)\rangle =\right|\Phi_{\tilde{m}}\rangle =\sum {\tilde{c}}_{n}|\Psi_{n}\rangle$. Thus, the wave function starts to coherently evolve according to the time-dependent Schrödinger equation $\Psi \left(t\right)=\sum_{n}{\tilde{c}}_{n}e^{-iE_{n}t}|\Psi_{n}\rangle$, where this and the following expressions are in atomic units. Representing the eigenstates via the basis states, the time evolution of the wave function can be expressed

(5) $$\begin{array}{c} |\Psi \left(t\right)\rangle =\sum_{n,m}a_{nm}e^{-iE_{n}t}|\Phi_{m}\rangle , \end{array}$$

where $a_{nm}=c_{n}^{\tilde{m}*}c_{n}^{m}$. We obtained $a_{nm}$ by connecting the coefficients ${\tilde{c}}_{n}$ to the coefficients $c_{n}^{m}$ and considering the initial condition at t=0. It holds $\sum_{n}c_{n}^{m}c_{n}^{\tilde{m}*}=\delta_{m,\tilde{m}}$ for the orthonormal basis, which results in ${\tilde{c}}_{n}=c_{n}^{\tilde{m}*}$.

The expression in Equation (5) describes the exciton transport right after the excitation: an exciton is localized at the site $\tilde{m}$ at time t=0 and gets delocalized over several molecules due to intersite coupling *V*. Exciton transport is a coherent process as long as molecular vibrations do not start destroying it. It has been heavily debated, whether coherent processes during exciton transport in molecular systems can survive for several hundred femtoseconds [3,7,8,9,10,11]. The assumption that coherence lasts for at least 20 fs is in any case reasonable [10]. We describe the pump-probe experiment for time delays up to 20 fs and neglect incoherent processes.

### 2.2. Interaction with an Ultrashort X-ray Probe Pulse

We assume that the molecular aggregate is probed by an ultrashort X-ray probe pulse with the Gaussian-shaped electric field

(6) $$\begin{array}{c} E\left(r,t\right)=\epsilon E_{0}e^{-\frac{2\ln2{\left(t-t_{p}\right)}^{2}}{\tau^{2}}}\cos\left(k\cdot r-\omega_{in}\left(t-t_{P}\right)\right) \end{array}$$

after the time, when Frenkel exciton dynamics has been launched. Here, $t_{p}$ is the delay time between the pump and the probe pulse, $\omega_{in}$ is the central energy, k is the wave vector, ϵ is the polarization of the X-ray pulse and τ is the probe-pulse duration. The choice of the probe-pulse duration depends on the characteristic time scale of coherent electron dynamics. The time evolution of any time-dependent observable $\langle \hat{O}\rangle =\langle \Psi \left(t\right)|\hat{O}|\Psi \left(t\right)\rangle$ of the Frenkel exciton system comprises harmonic oscillations with periods $2\pi /(E_{n}-E_{n^{\prime }})$, where $n\neq n^{\prime }$. The shortest possible beating period is given by the maximum splitting of the eigenvalues of the Frenkel Hamiltonian. It is equal to 4V for the chain of equivalent molecules. Thus, the duration of the probe pulse τ must be shorter than π/(2V) to resolve the finest details of exciton dynamics.

The total Hamiltonian in the presence of the X-ray pulse is given

(7) $$\begin{array}{c} \hat{H}={\hat{H}}_{0}-\hat{d}E\left(r,t\right), \end{array}$$

where $\hat{d}$ is the dipole operator. Time-resolved probability of X-ray absorption from a general coherently evolving electronic system has been derived in Ref. [35] by means of the first-order time-dependent perturbation theory. Applying these results in Appendix A.1, we obtain the absorption cross section as a function of pump-probe time delay and X-ray energy for our system

(8) $$\begin{array}{c} \sigma \left(t_{p}\right)=\frac{4\pi^{2}}{\omega_{in}}\sum_{F}{\left|\sum_{m}\Omega_{F}^{m}\left(t_{p}\right)\langle \Phi_{F}|e^{ik\cdot r}\epsilon \cdot \hat{d}|\Phi_{m}\rangle \right|}^{2} \end{array}$$

with the function

(9) $$\begin{array}{c} \Omega_{F}^{m}\left(t_{p}\right)=\sqrt{\frac{\pi \tau^{2}}{8\ln2}}E_{0}\sum_{n}a_{nm}e^{-iE_{n}t_{p}}e^{-\frac{{\left(E_{F}-E_{n}-\omega_{in}\right)}^{2}\tau^{2}}{8\ln2}}. \end{array}$$

Here, the sum runs over all possible final states $|\Phi_{F}\rangle$ with eigenenergies $E_{F}$ that can be reached after X-ray absorption. $|\Phi_{F}\rangle$ are eigenstates of a general electronic Hamiltonian, but not of the Frenkel Hamiltonian. The electronic Hamiltonian can be described by the Frenkel Hamiltonian only before the X-ray absorption step. After X-ray absorption, the system is in a core-excited state, which is beyond the Frenkel exciton model.

According to Equation (8), the absorption cross section is determined by the series of peaks with time-dependent amplitudes. The bandwidth and the position of the peaks are encoded in the Gaussian functions $e^{-\left({\left(E_{F}-E_{n}-\omega_{in}\right)}^{2}\tau^{2}\right)/\left(8\ln2\right)}$. The bandwidth of the peaks is inversely proportional to the X-ray probe pulse duration τ. Here, we assumed that the pulse duration is considerably shorter than the shortest beating period of the coherent exciton dynamics. In the spectral domain, this automatically implies that the bandwidth of the probe pulse and, subsequently, the bandwidth of the spectral peaks is considerably larger than the largest energy splitting between the eigenstates of the Frenkel Hamiltonian. The difference in the spectral positions due to different energies $E_{n}$ cannot be distinguished and we can substitute a mean value of the Frenkel Hamiltonian eigenenergies 〈E〉 for any $E_{n}$ in Equation (9).

The time-dependence of the amplitude of the peaks are governed by the terms $a_{nm}e^{-iE_{n}t_{p}}$, which already indicates that time-resolved cross section is sensitive to the coherent Frenkel exciton dynamics. The amplitudes are also determined by the matrix elements of the X-ray transitions $\langle \Phi_{F}|e^{ik\cdot r}\epsilon \cdot \hat{d}|\Phi_{m}\rangle$. In the following, we will analyze these matrix elements by considering different possible final states after X-ray absorption $|\Phi_{F}\rangle$.

## 3. Results

In X-ray absorption spectroscopy, the energy of the X-ray pulse is tuned to the binding energy of a certain core orbital. The binding energies of core orbitals strongly vary depending on the type of the orbital and atomic species. They can be even considerably different for the same atomic species being in different chemical environments. Atomic specificity and chemical sensitivity makes this technique attractive for the study of a structure and its dynamics.

Let’s consider that an X-ray probe pulse is resonant to an orbital *j* of a certain atomic type. Core orbitals are strongly localized around the corresponding atom. That is why we can characterize every final many-body state *F* by the position of a core hole created after X-ray absorption. The electron from a localized orbital is excited to a valence orbital that is delocalized. Thus, a many-body final state can be characterized by a hole in a certain atom *j* located at a molecule *m* and a type of an excitation in the valence state. There can be different atoms *j* of the same type located on the same molecule. For example, the X-ray pulse may be tuned to the binding energy of the 1 s orbital of a Carbon atom and there can be several Carbon atoms in a molecule. Thus, the summation over final states will always imply the summation over orbitals *j* and molecules *m*, where the core hole can be located. In the following, we will discuss possible final states and show how X-ray absorption cross section depends on them.

### 3.1. X-ray Transitions below Fermi Level

Let us now assume that we tune the energy of the X-ray pulse to match the energy of an electronic transition from a core orbital to a highest occupied molecular orbital (HOMO). If the system were in the ground state, all HOMO orbitals of all molecules would be doubly occupied and such a transition would be forbidden. But since there is an exciton in the system such a transition becomes possible. The electron excited from the core orbital occupies the vacancy in the HOMO orbital. The final core-excited state has all HOMO orbitals doubly occupied, one hole in a core orbital and one electron above the Fermi level, in a LUMO orbital.

**Figure 2:**
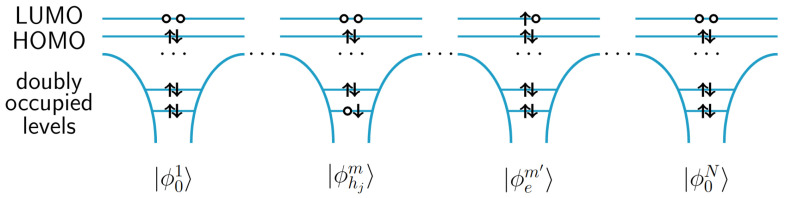
A many-body basis state $|\Phi_{F_{m,j,m^{\prime }}}\rangle$, in which a core electron occupied a vacant HOMO orbital after absorption of an X-ray photon.

We can represent a final state in the basis of many-body core-excited states of the type

(10) $$\begin{array}{c} |\Phi_{F_{m,j,m^{\prime }}}\rangle =|\phi_{0}^{1}\rangle \ldots ⊗|\phi_{h_{j}}^{m}\rangle \ldots ⊗|\phi_{e}^{m^{\prime }}\rangle ⊗\ldots |\phi_{0}^{N}\rangle , \end{array}$$

where $|\phi_{h_{j}}^{m}\rangle$ denotes the state of a molecule *m* with a hole in the core orbital located at the atom *j* as shown in Figure 2. $|\phi_{e}^{m^{\prime }}\rangle$ denotes the state of the molecule $m^{\prime }$, all HOMO orbitals of which are doubly occupied and one LUMO is singly occupied. The core holes of the final states are strongly localized orbitals. But the analogously to the Frenkel exciton model, LUMO orbitals of the final states maybe delocalized due to coupling between nearest-neighbour molecules. This means that the basis states $|\Phi_{F_{m,j,m^{\prime }}}\rangle$ may not be the eigenstates of the electronic Hamiltonian.

We assume that the states with a different location of a core hole have the same energy. We also assume that the maximum possible difference between the eigenenergies of the states $|\Phi_{F_{m,j,n}}\rangle$ and $|\Phi_{F_{m,j,n^{\prime }}}\rangle$ is similar to the energy difference between the eigenstates of Frenkel Hamiltonian. Thus, the variation of the final-state energies $E_{F}$ would be negligible within the bandwidth of the probe pulse and we substitute $\langle E_{F}\rangle$ for any $E_{F}$. In this case, the substitution of the basis core-excited states for the final states in the expression for the time-resolved cross section in Equation (8) would not change the result (cf. Appendix A.2). We perform this substitution, since it facilitates the analysis.

The derivation of the cross section can now be reduced to the analysis of the matrix elements between the basis states of Frenkel Hamiltonian and the basis core-excited states, $\langle \Phi_{F_{m,j,m^{\prime }}}|e^{ik\cdot r}\epsilon \cdot \hat{d}|\Phi_{m^{\prime \prime }}\rangle$. The electron in the LUMO orbital in $|\Phi_{F_{m,j,m^{\prime }}}\rangle$ and $|\Phi_{m^{\prime \prime }}\rangle$ must be on the same site before and after the X-ray transition:

(11) $$\begin{array}{cc} \; & \left|\phi_{0}^{1}\rangle \ldots ⊗\right|\phi_{0}^{m}\rangle \ldots ⊗|\phi_{*}^{m^{\prime }}\rangle ⊗\ldots |\phi_{0}^{N}\rangle \end{array}$$

(12) $$\begin{array}{cc} \; & \to |\phi_{0}^{1}\rangle \ldots ⊗|\phi_{h_{j}}^{m}\rangle \ldots ⊗|\phi_{e}^{m^{\prime }}\rangle ⊗\ldots |\phi_{0}^{N}\rangle , \end{array}$$

which means that only matrix elements

(13) $$\begin{array}{c} \langle \Phi_{F_{m,j,m^{\prime }}}|e^{ik\cdot r}\epsilon \cdot \hat{d}|\Phi_{m^{\prime }}\rangle \end{array}$$

are relevant for our further analysis of the cross section.

In the Hartree-Fock approximation, the transition matrix element would reduce to the integral involving the core orbital $\varphi_{c}\left(r-R_{m,j}\right)$ located at an atom *j* at the molecule *m*, where $R_{m,j}$ is the position of the orbital, and the HOMO orbital $\varphi_{H}\left(r-R_{m^{\prime }}\right)$ of the molecule $m^{\prime }$ centered at $R_{m^{\prime }}$

(14) $$\begin{array}{c} \langle \Phi_{F_{m,j,m^{\prime }}}\left|e^{ik\cdot r}\epsilon \cdot \hat{d}\right|\Phi_{m^{\prime }}\rangle =e^{ik\cdot R_{m,j}}\int d^{3}r\varphi_{c}\left(r-R_{m,j}\right)\left(\epsilon \cdot r\right)\varphi_{H}\left(r-R_{m^{\prime }}\right). \end{array}$$

Here, we use the standard assumption that the wave length of the X-ray pulse is larger than the spatial extend of the core orbital [36], which allowed us to factor out $e^{ik\cdot r}$ from the integral. If the orbitals $\varphi_{H}\left(r-R_{m}\right)$ and $\varphi_{c}\left(r-R_{m^{\prime },j}\right)$ do not have any spatial overlap, the integral above is zero. Thus, the most intuitive assumption to make is that an electron from a core orbital of the molecule *m* can be excited only to a HOMO orbital of the same molecule

(15) $$\begin{array}{c} \langle \Phi_{F_{m,j,m^{\prime }}}\left|e^{ik\cdot r}\epsilon \cdot \hat{d}\right|\Phi_{m^{\prime }}\rangle =e^{ik\cdot R_{m,j}}\delta_{m,m^{\prime }}d_{j}, \end{array}$$

where $d_{j}=\int d^{3}r\varphi_{c}\left(r-R_{m,j}\right)\left(\epsilon \cdot r\right)\varphi_{H}\left(r-R_{m}\right)$. $d_{j}$ does not depend on the position of the molecule. We assume that the orbitals are real functions, which means $d_{j}$ is also real.

This considerably simplifies the expression for the cross section, which becomes a time-independent function as we derive in Appendix A.3

(16) $$\begin{array}{cc} \sigma (t_{p})= & \frac{\pi^{3}E_{0}^{2}\tau^{2}}{2\ln2\omega_{in}}e^{-\frac{{\left(\langle E_{F}\rangle -\langle E\rangle -\omega_{in}\right)}^{2}\tau^{2}}{4\ln2}}\sum_{j}d_{j}^{2}. \end{array}$$

Our assumptions about the transition matrix element led to the appearance of $\delta_{n,n^{\prime }}$ in the expression for the cross section, which cancelled out any time-dependent terms. The appearance of $\delta_{n,n^{\prime }}$ means that there is no final state, which can be reached from two different eigenstates of Frenkel Hamiltonian. The interference between different transition channels got forbidden and time-dependent information got lost.

Let us revisit the transition matrix element and the integral in Equation (14). We made an assumption that only an orbital on a molecule *m* can have a spatial overlap with a core orbital located on the same molecule *m*. Actually, this should not be the case for Frenkel exciton model. The main assumption of this model is that molecules are coupled via the coupling constant *V*. This implies that a HOMO and the lowest unoccupied molecular orbital (LUMO) of a molecule must extend to its next-neighbour molecules and we can assume

(17) $$\begin{array}{c} \langle \Phi_{F_{m,j,m^{\prime }}}\left|e^{ik\cdot r}\epsilon \cdot \hat{d}\right|\Phi_{m^{\prime }}\rangle =e^{ik\cdot R_{m,j}}\left(\delta_{m,m^{\prime }}d_{j}+\delta_{m,m^{\prime }\pm 1}{\bar{d}}_{j}\right), \end{array}$$

where ${\bar{d}}_{j}=\int d^{3}r\varphi_{c}\left(r-R_{m,j}\right)\left(\epsilon \cdot r\right)\varphi_{H}\left(r-R_{m\pm 1}\right)$, but ${\bar{d}}_{j}$ is considerably smaller than $d_{j}$.

The time-dependent absorption cross section then becomes (cf. Appendix A.3)

(18) σ(tp)=π3E02τ22ln2ωine−(EF−〈E〉−ωin)2τ24ln2×∑jdj2+2djd¯jRe∑m,n,n′an′m+1∗anm+an′m−1∗anme−i(En−En′)tp.

The first term in the squared brackets in this expression is time-independent. The second term in the squared brackets contains time-dependent terms with $n\neq n^{\prime }$ and provides time-resolved information about the dynamics. The assumption that a core electron of a molecule can be excited to the delocalized HOMO orbital of a neighbouring molecule with some probability made the interference between different transition channels possible. In the Section 3.3, we will connect properties of exciton dynamics to the time-resolved part of the cross section.

### 3.2. X-ray Transitions above Fermi Level

**Figure 3:**
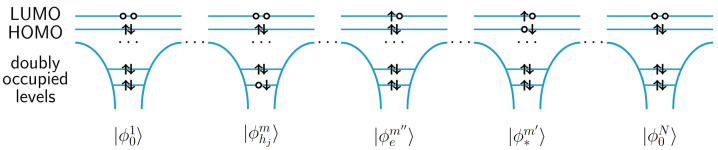
A many-body basis state $|\Phi_{F_{m,j,m^{\prime },m^{\prime \prime }}}\rangle$, in which a core electron occupied a vacant LUMO orbital after absorption of an X-ray photon.

Let us now analyze the case, when the X-ray pulse is resonant to the energy of transitions to states above Fermi level, i.e., into LUMO orbitals. A final state after absorption would be a doubly excited state with two holes and two electrons in the LUMO orbitals. In a simple picture, where correlation effects do not play a considerable role, we can consider possible final states in the basis of states

(19) $$\begin{array}{c} \left|\phi_{0}^{1}\rangle \ldots ⊗\right|\phi_{h_{j}}^{m}\rangle \ldots ⊗|\phi_{e}^{m^{\prime }}\rangle \ldots ⊗|\phi_{h}^{m^{\prime \prime }}\rangle \ldots ⊗|\phi_{e}^{m^{\prime \prime \prime }}\rangle \ldots ⊗|\phi_{0}^{N}\rangle , \end{array}$$

where one hole is in the core orbital located at atom *j* at molecule *m*, one hole is in the HOMO orbital of a molecule $m^{\prime \prime }$, one electron is in the LUMO orbital of molecule $m^{\prime }$ and one electron is in the LUMO orbital of molecule $m^{\prime \prime \prime }$. If we ignore correlation effects, the HOMO-LUMO excitation at the molecule $m^{\prime }$ is not destroyed after the transition of a core electron into the manifold of unoccupied states (see Figure 3). This means that X-ray absorption transitions are possible only for the final states with $m^{\prime }=m^{\prime \prime }$ or $m^{\prime \prime }=m^{\prime \prime \prime }$

(20) $$\begin{array}{cc} \; & \left|\phi_{0}^{1}\rangle \ldots ⊗\right|\phi_{*}^{m^{\prime }}\rangle ⊗\ldots |\phi_{0}^{N}\rangle \\ \; & \to |\Phi_{F_{m,j,m^{\prime },m^{\prime \prime }}}\rangle =|\phi_{0}^{1}\rangle \ldots ⊗|\phi_{h_{j}}^{m}\rangle \ldots ⊗|\phi_{e}^{m^{\prime \prime }}\rangle \ldots ⊗|\phi_{*}^{m^{\prime }}\rangle \ldots ⊗|\phi_{0}^{N}\rangle . \end{array}$$

Thus, only matrix elements $\langle \Phi_{F_{m,j,m^{\prime },m^{\prime \prime }}}|e^{ik\cdot r}\epsilon \cdot \hat{d}|\Phi_{m^{\prime }}\rangle$ matter for the cross section.

With the same arguments as in the previous subsection, the transition matrix element becomes

(21) $$\begin{array}{c} \langle \Phi_{F_{m,j,m^{\prime },m^{\prime \prime }}}\left|e^{ik\cdot r}\epsilon \cdot \hat{d}\right|\Phi_{m^{\prime }}\rangle =e^{ik\cdot R_{m,j}}\left(\delta_{m,m^{\prime \prime }}d_{j}^{\prime }+\delta_{m,m^{\prime \prime }\pm 1}{\bar{d}}_{j}^{\prime }\right), \end{array}$$

where $d_{j}^{\prime }$ and ${\bar{d}}_{j}^{\prime }$ describe the transition matrix element from a core electron located at the atom *j* of the molecule *m* into the LUMO orbital of the same molecule *m* and into the LUMO orbital of the next-neighbour molecules m±1, respectively.

We derive the time-resolved cross section for resonant transitions above the Fermi level In Appendix A.4 and obtain that it does not provide any time-resolved information about the dynamics

(22) $$\begin{array}{cc} \sigma (t_{p})= & \frac{\pi^{3}E_{0}^{2}\tau^{2}}{2\ln2\omega_{in}}e^{-\frac{{\left(\langle E_{F}\rangle -\langle E\rangle -\omega_{in}\right)}^{2}\tau^{2}}{4\ln2}}\sum_{j}\left[N{d_{j}^{\prime }}^{2}+\left(2N-2\right){{\bar{d}}_{j}^{\prime }}^{2}\right]. \end{array}$$

This happens even despite the fact that we allowed the transitions of core electrons to the nearest-neighbour molecules as in the previous case. The reason for this result is again the absence of interference between different transitions channels. It is not possible to reach the same many-body final state by X-ray transitions from different eigenstates involved in the dynamics and the temporal interference becomes lost.

The cross section for X-ray transitions above Fermi level is ∼N times larger than the cross section for resonant transitions below the Fermi level. This is because there are *N* unoccupied LUMO orbitals per basis state of the Frenkel exciton model above the Fermi level, whereas there are only one vacant HOMO orbital below the Fermi level. Thus, the time-independent contribution to the cross section would be considerably larger than the time-resolved one. Fortunately, one can exclude the transitions above the Fermi level by tuning the X-ray photon energy to be lower and match only the resonance of the transition to a HOMO orbital. One indeed needs to make sure that the probability of the transitions above the Fermi level does not start to become considerable due to a broad spectral bandwidth of the probe pulse.

We analyzed the transitions above the Fermi level ignoring correlation effects, which may start to play a role due to a doubly excited state of the chain. This may provide some correction to the expression for the cross section in Equation (22) and may even allow for some interference between transition channels. However, such an interference term, if any, would be a small correction to a large time-independent background proportional to *N* and a resulting time-dependent contribution would be negligible.

### 3.3. Connection of the Time-Resolved X-ray Absorption cross Section to Frenkel Exciton Dynamics

The density matrix of the Frenkel exciton system is given by

(23) $$\begin{array}{c} \hat{\rho }\left(t\right)=\left|\Psi \left(t\right)\rangle \langle \Psi \left(t\right)\right| \end{array}$$

by the definition. Using the expression in Equation (5), we can represent it in the site basis states

(24) $$\begin{array}{cc} \hat{\rho }\left(t\right) & =\sum_{m,m^{\prime }}\rho_{m,m^{\prime }}\left(t\right)|\Phi_{m}\rangle \langle \Phi_{m^{\prime }}| \end{array}$$

with

(25) $$\begin{array}{cc} \rho_{m,m^{\prime }}\left(t\right) & =\sum_{n,n^{\prime }}a_{n^{\prime }m^{\prime }}^{*}a_{nm}e^{-i(E_{n}-E_{n^{\prime }})t}. \end{array}$$

The terms $\rho_{m,m^{\prime }}\left(t\right)$ are the relevant property of the Frenkel exciton dynamics, since it determines coherences between different sites. The temporal evolution of the exciton density is determined by the elements of the density matrix (exciton density is a function of space and is not to be confused with the density matrix). The exciton density consists of the spatial distribution localized around the molecules and factored by the populations $\rho_{m,m}\left(t\right)$ and the spatial distribution in the intersite region and factored by 2Re(ρm,m′). The spatial distribution in the intersite region exists, if the orbitals of sites *m* and $m^{\prime }$ have spatial overlap.

Let us consider the observable ${\hat{O}}_{m,m^{\prime }}={\hat{c}}_{m^{\prime }}^{†}{\hat{h}}_{m^{\prime }}^{†}{\hat{c}}_{m}{\hat{h}}_{m}+c.c.$ that describes the degree of exciton delocalization between sites *m* and $m^{\prime }$, if $m\neq m^{\prime }$. Here, ${\hat{c}}_{m(m^{\prime })}$ and ${\hat{h}}_{m(m^{\prime })}$ are electron and hole annihilation operators at site *m* ($m^{\prime }$) that we used to define the Frenkel Hamiltonian in Section 2.1. The expectation value of ${\hat{O}}_{m,m^{\prime }}$ is given by

(26) 〈O^m,m′〉=2SReρm,m′(t)(δm,m′+1+δm,m′−1),

where *S* is the overlap integral between site basis states $\langle \Phi_{m^{\prime }}|{\hat{c}}_{m^{\prime }}^{†}{\hat{h}}_{m^{\prime }}^{†}{\hat{c}}_{m}{\hat{h}}_{m}|\Phi_{m}\rangle$, which is nonzero only for $m^{\prime }=m\pm 1$. $\langle {\hat{O}}_{m,m^{\prime }}\rangle$ is the measure of the spatial contribution of the intersite region between molecules *m* and $m^{\prime }$ to the total Frenkel-exciton density. Using observable $\langle {\hat{O}}_{m,m^{\prime }}\rangle$, we can define the degree of exciton intersite delocalization as

(27) $$\begin{array}{c} h_{\mathrm{inter}}\equiv \sum_{m,m^{\prime }}\langle {\hat{O}}_{m,m^{\prime }}\rangle , \end{array}$$

which becomes

(28) hinter(t)=2S∑m,n,n′Rean′m+1∗anm+an′m−1∗anme−i(En−En′)t.

The time-dependent part of the X-ray absorption cross section in Equation (18) follows exactly the time evolution of the intersite delocalization degree $h_{\mathrm{inter}}\left(t\right)$.

To illustrate our results, we model coherent Frenkel exciton dynamics and attosecond X-ray absorption spectra of a homoaggregate of eleven equidistantly aligned molecules. We select the intersite coupling V=0.22 eV and the Coulomb energy of an electron-hole pair localized on a single site U=2.7 eV, which are typical parameters for Frenkel exciton [37,38,39]. We assume that the molecule in the middle of the chain got excited at time zero, i.e., $\tilde{m}=6$.

**Figure 4:**
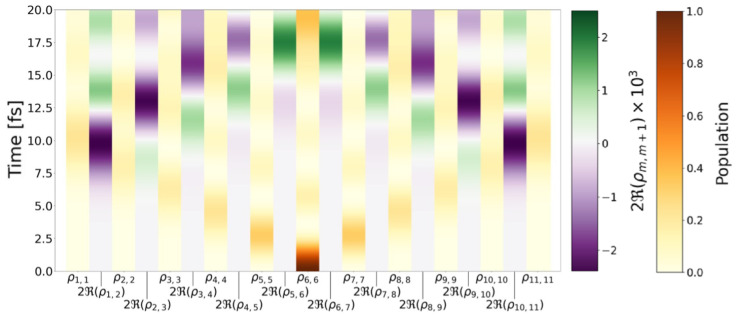
Populations $\rho_{m,m}$ for all molecules in the chain and the real part of the coherences 2Re(ρm,m+1) for all nearest neighbours depending on time.

Figure 4 illustrates the time evolution of the site populations, $\rho_{m,m}\left(t\right)$, and of the real part of the coherences 2Re(ρm,m+1(t)). We find that Re(ρm,m+1(t)) are negligible first 5 fs, whereas Im(ρm,m+1(t)) are considerable during this time. The imaginary part of density matrix elements determine the time-dependent electron current density. This means that the electron current density between sites is considerable first 5 fs and the exciton density starts to fill the intersite regions after 5 fs.

The maximum splitting between eigenenergies of Frenkel Hamiltonian is given by 4V=0.88 eV, which means that the shortest beating period of the coherent time evolution of the Frenkel excitons is 4.7 fs. We select the X-ray probe pulse duration τ=500 as to be short enough to resolve the finest details of the dynamics. We assume that the X-ray probe pulse is resonant with the energy of the excitation of 1 s Carbon orbital to the HOMO orbital, which is approximately 283 eV.

**Figure 5:**
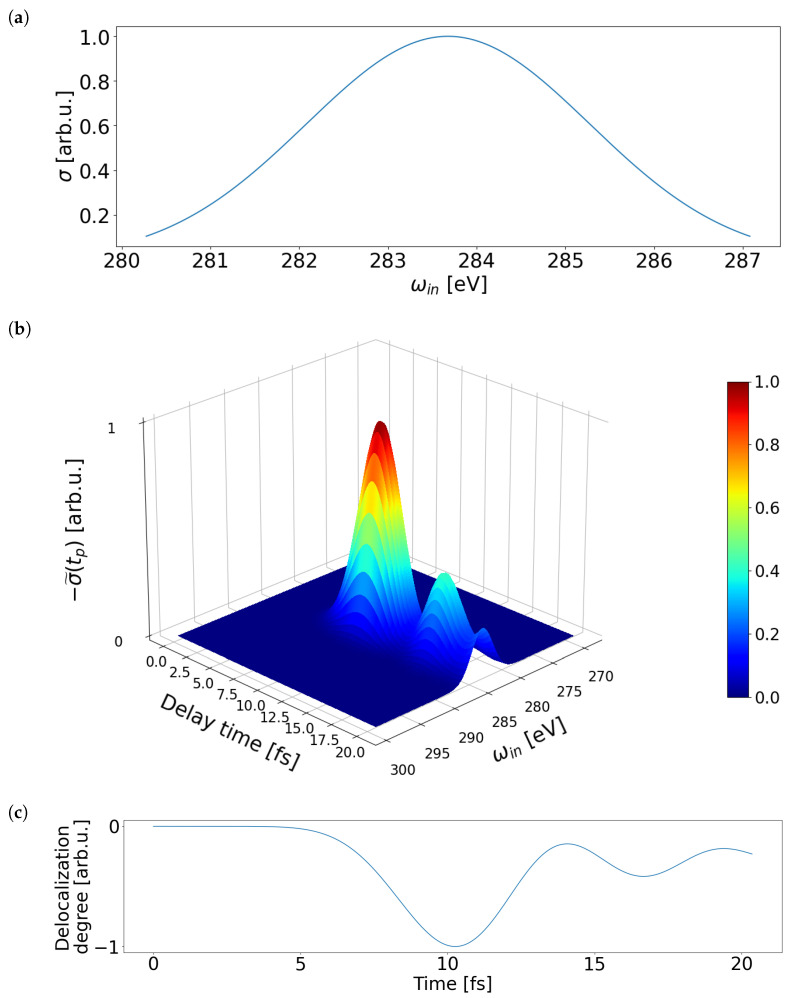
(**a**) Normalized cross section at the time delay of 500 as as a function of energy. X-ray probe pulse is assumed to be resonant with the transition energy of an electron from the 1 s orbital of Carbon into outermost orbitals below Fermi level. (**b**) Normalized time evolution of the change in the cross section after the delay time of 500 as, $\tilde{\sigma }\left(t_{p}\right)=\sigma \left(t_{p}\right)-\sigma \left(0.5\;\mathrm{fs}\right)$, as a function of energy and time. (**c**) Time evolution evolution of the normalized delocalization degree $h_{\mathrm{inter}}\left(t\right)$.

We assume that $\sum_{j}d_{j}{\bar{d}}_{j}$ is ten times smaller as $\sum_{j}d_{j}^{2}$ and calculate the cross section as a function of X-ray probe pulse central energy and pump-probe time delay. Figure 5a shows the normalized total cross section at the delay time of 500 as. The cross section is given by a Gaussian peak with FWHM of 3.8 eV centered at ∼284 eV as shown in Figure 5a. The position of the peak is constant, but the amplitude is time-dependent.

The time evolution of the change in the cross section after the delay time of 500 as, $\tilde{\sigma }\left(t_{p}\right)=\sigma \left(t_{p}\right)-\sigma \left(0.5\;\mathrm{fs}\right)$, as a function of energy and time is shown in Figure 5b. The change is negative for our system during the considered pump-probe time delay of 0.5–20 fs. The relative change in the cross section $\tilde{\sigma }\left(t_{p}\right)/\sigma \left(t_{p}\right)$ depends only on time and not on energy and is about 1% of the total signal. Figure 5c shows the time evolution of $h_{\mathrm{inter}}\left(t\right)$ obtained with Equation (28). The time evolution of the X-ray signal precisely follows the delocalization degree. Extraction of exciton delocalization is relevant for understanding exciton transport in molecular aggregates [40]. We obtained that attosecond X-ray absorption spectroscopy provides an access to this property in real time.

We can reveal even more details about Frenkel excitons from the X-ray absorption signal. Nonzero signal for Carbon K-edge reveals the *p*-type structure of the HOMO orbitals, where the hole of Frenkel exciton resides. Another encoded detail is about the bonds connecting the neighbouring molecules. The occurrence of the time-dependent part of the signal is governed by a nonzero transition matrix element ${\bar{d}}_{j}$. ${\bar{d}}_{j}$ is nonzero, if there is a spatial overlap between orbitals of nearest-neighbour molecules. Thus, it carries information about the bonds responsible for intersite coupling.

## 4. Conclusions

In this article, we explored novel capabilities of attosecond X-ray pulses by analyzing how they can probe coherent Frenkel exciton dynamics in molecular aggregates. We focused on Frenkel exciton transport for up to 20 fs, when coherent processes dominate the dynamics. We investigated what attosecond X-ray absorption can reveal about coherent transport. We assumed that the X-ray probe pulse duration is short enough to resolve the shortest beating period of the coherent dynamics. In energy domain, this means that widths of the spectral peaks are considerably broader than energy splittings of electronic eigenstates involved in the dynamics.

We analyzed two different types of X-ray transitions. One case is then the X-ray pulse is resonant to the excitation of an electron from a core orbital to the orbitals below Fermi level, where a hole of the exciton resides. The second case is the transitions to the orbitals above Fermi level, where the electron of the exciton resides. In both cases the spectrum would be formed by a broad spectral peak centered at the central energy of the X-ray pulse. But since the X-ray pulse would be tuned to two different energies, the both peaks would be spectroscopically distinguishable.

Interference effects are necessary for a signal to be time-dependent. The interference of the signal is created, when the same final core-excited state can be reached from several eigenstates involved in the dynamics. We have shown that it is not possible to reach the same final core-excited state from two different eigenstates of Frenkel Hamiltonian for resonant transitions to states above Fermi level. Thus, the part of the X-ray spectrum formed by the peak corresponding to transitions above Fermi level would be simply time-independent and would not carry any information about the coherent dynamics.

The part of the signal corresponding to transitions below Fermi level does carry time-resolved information. The time evolution of the peak amplitude precisely follows the time evolution of the degree of exciton delocalization. The degree of exciton delocalization gives the information about how much of the exciton density is distributed in the intersite region.

Exciton delocalization is at the core of electronic energy transfer of molecular excitons [40]. Electronic energy transfer is the dynamical regime in which pure coherent evolution competes with dissipative evolution in contrast to transfer by incoherent hopping jumps in Förster regime. Electronic energy transfer accompanies photosynthetic processes that result in highly efficient light-energy conversion [41]. Understanding the principles of highly efficient light harvesting in photosynthesis is crucial for design of efficient organic solar cells that also involve molecular excitons for energy transfer. Measuring exciton delocalization will provide the insight into site superposition of excitations present during electronic energy transfer of molecular excitons. Access to this property on attosecond time scale will additionally reveal the role of pure electronic degrees of freedom for electronic energy transfer.

An optical pulse can also be used as an attosecond probe of electron dynamics. But there is one key difference of an optical probe to an X-ray probe. Optical pulses induce electronic transitions from delocalized orbitals to delocalized orbitals, and X-ray pulses induce transitions of electrons from localized orbitals to delocalized orbitals. This specifics of X-ray transitions obviously leads to atomic sensitivity. But other essential advantage for probing exciton transport is that transitions involving localized core states disentangle the degree of delocalization in valence states and map it into the time-resolved part of the X-ray signal.

## Data Availability

Data sharing not applicable.

## Abbreviations

The following abbreviations and notations are used in this manuscript:

HOMO	Highest occupied molecular orbital
LUMO	Lowest unoccupied molecular orbital
$E_{0}$	Amplitude of the electric field of a probe pulse
$t_{p}$	Pump-probe time delay
$\omega_{in}$	Central frequency of a probe pulse
k	Wave vector of a probe pulse
ϵ	Polarization vector of a probe pulse
τ	Probe pulse duration
$\tilde{m}$	Index for the initially excited molecule
*j*	Index for the atom *j*
*m*	Index for the molecule *m*
U	Coulomb energy of an electron-hole pair localized on a single site
V	Coupling between nearest-neighbor molecules
$h_{inter}$	Degree of the exciton intersite delocalization
${\tilde{c}}_{n}$	Expansion coefficients of the representation of the time-dependent wave function as eigenstates
$c_{n}^{m}$	Expansion coefficients of the representation of the eigenstates of Frenkel Hamiltonian as basis states
$a_{nm}$	Expansion coefficients of the solution of the time-dependent Schrödinger equation
$d_{j}$	Transition matrix element between core electron orbital of the atom j of molecule m and
	HOMO of the same molecule
${\bar{d}}_{j}$	Transition matrix element between core electron orbital of the atom j of the molecule m and
	HOMO of the next-neighbor molecules
$d_{j}^{\prime }$	Transition matrix element between core electron orbital of the atom j of the molecule m and
	LUMO of the same molecule
${\bar{d}}_{j}^{\prime }$	Transition matrix element between core electron of the atom j of the molecule m and
	LUMO of the next-neighbor molecules
$|\phi_{0}^{m}\rangle$	Ground state of the molecule *m*
$|\Psi_{0}\rangle$	Ground state of a chain of molecules
$|\phi_{*}^{m}\rangle$	State which corresponds to an electron-hole pair located at the site *m*
$|\Phi_{m}\rangle$	Basis state of Frenkel Hamiltonian
$|\Psi_{n}\rangle$	Eigenstate of Frenkel Hamiltonian
$|\phi_{h_{j}}^{m}\rangle$	State of the molecule *m* with core hole at atom *j*
$|\phi_{e}^{m}\rangle$	State of the molecule *m* with one electron in LUMO
$|\phi_{h}^{m}\rangle$	State of the molecule *m* with hole in HOMO
$|\Phi_{F}\rangle$	Final state after absorption
$|\Phi_{F_{m,j,m^{\prime }}}\rangle$	Final state with a core hole at the atom *j* of the molecule *m* and an electron in LUMO of the molecule $m^{\prime }$
$|\Phi_{F_{m,j,m^{\prime },m^{\prime \prime }}}\rangle$	Final state with a core hole at the atom *j* of the molecule *m*, an electron-hole pair at the site $m^{\prime }$ and electron in LUMO at the site $m^{\prime \prime }$

## Appendix A

### Appendix A.1. Derivation of Time-Resolved X-ray Absorption cross Section

The absorption cross section within the first-order time-dependent perturbation theory is given by [35]

(A1) $$\begin{array}{c} \sigma \left(t_{p}\right)=\frac{4\pi^{2}}{\omega_{in}}\lim_{t_{0}\to -\infty }\sum_{F}{\left|\int_{t_{0}}^{+\infty }dt\langle \Phi_{F}|e^{i{\hat{H}}_{0}t}E\left(r,t-t_{p}\right)\hat{d}e^{-i{\hat{H}}_{0}t}|\Psi \left(t_{0}\right)\rangle \right|}^{2} \end{array}$$

Here, the sum runs over all possible final states $|\Phi_{F}\rangle$ with eigenenergies $E_{F}$ that can be reached after X-ray absorption. Substituting the time-dependent wave function represented in the basis of the Frenkel basis states in Equation (5), we obtain

(A2) $$\begin{array}{c} \sigma \left(t_{p}\right)=\frac{4\pi^{2}}{\omega_{in}}E_{0}^{2}\sum_{F}{\left|\int_{-\infty }^{+\infty }dt\langle \Phi_{F}|e^{iE_{F}t}e^{ik\cdot r}\left(\epsilon \cdot \hat{d}\right)e^{-\frac{2\ln2{\left(t-t_{p}\right)}^{2}}{\tau^{2}}}\left(\frac{e^{-i\omega_{in}\left(t-t_{p}\right)}}{2}\right)\sum_{n,m}a_{nm}e^{-iE_{n}t}|\Phi_{m}\rangle \right|}^{2}. \end{array}$$

Performing the integration over time, we obtain the expression for the time-resolved absorption cross section in Equation (8) in Section 2.2.

### Appendix A.2. Representation of the X-ray Absorption cross Section via Basis States

Let us represent the final states in Equation (8) via basis states $|\Phi_{F_{m,j,m^{\prime }}}\rangle$ described in Section 3.1: $|\Phi_{F_{m,j,n}}\rangle =\sum_{m^{\prime }}b_{n,m^{\prime }}|\Phi_{F_{m,j,m^{\prime }}}\rangle$. Since a core orbital is strongly localized, each final eigenstate can be characterized by the position of the core hole in the atom *j* of a molecule *m*. The index *n* refers to a delocalized valence orbital. We assume that the final eigenstates $|\Phi_{F_{m,j,n}}\rangle$ and $|\Phi_{F_{m^{\prime },j,n}}\rangle$ with a different location of a core hole have the same energy. We also assume that the maximum possible difference between the eigenenergies of the states $|\Phi_{F_{m,j,n}}\rangle$ and $|\Phi_{F_{m,j,n^{\prime }}}\rangle$ is similar to the energy difference between the eigenstates of Frenkel Hamiltonian. Thus, the variation of the final-state energies $E_{F}$ would be negligible within the bandwidth of the probe pulse and we substitute $\langle E_{F}\rangle$ for any $E_{F}$. We obtain that the expression for the cross section does not depend on the representation of the final states:

(A3) $$\begin{array}{cc} \sigma (t_{p})= & \frac{4\pi^{2}}{\omega_{in}}\sum_{m,j,n}{\left|\langle \sum_{m^{\prime }}b_{n,m^{\prime }}^{*}\Phi_{F_{m,j,m^{\prime }}}|e^{ik\cdot r}\epsilon \cdot \hat{d}|\tilde{\Phi }\rangle \right|}^{2}\\ = & \frac{4\pi^{2}}{\omega_{in}}\sum_{m,j,n}\sum_{m^{\prime },{\tilde{m}}^{\prime }}b_{n,m^{\prime }}^{*}b_{n,{\tilde{m}}^{\prime }}\langle \Phi_{F_{m,j,m^{\prime }}}|e^{ik\cdot r}\epsilon \cdot \hat{d}|\tilde{\Phi }\rangle \langle \tilde{\Phi }|e^{ik\cdot r}\epsilon \cdot \hat{d}|\Phi_{F_{m,j,{\tilde{m}}^{\prime }}}\rangle \\ = & \frac{4\pi^{2}}{\omega_{in}}\sum_{m,j,m^{\prime }}{\left|\langle \Phi_{F_{m,j,m^{\prime }}}|e^{ik\cdot r}\epsilon \cdot \hat{d}|\tilde{\Phi }\rangle \right|}^{2} \end{array}$$

where we used the notation $\sum_{m^{\prime \prime }}\Omega_{F}^{m^{\prime \prime }}\left(t_{p}\right)|\Phi_{m^{\prime \prime }}\rangle =|\tilde{\Phi }\rangle$ and that $\sum_{n}b_{n,m^{\prime }}^{*}b_{n,{\tilde{m}}^{\prime }}=\delta_{m^{\prime },{\tilde{m}}^{\prime }}$, since they are coefficients of an orthonormal basis.

### Appendix A.3. Derivation of X-ray Absorption cross Section for Transitions below Fermi Level

Let us consider the time-resolved absorption cross section with the two approximations considered in Section 3.1: X-ray energy is resonant to the transition to a HOMO orbital and the HOMO orbital of a molecule does not have any spatial overlap with a core orbital of an atom located at a different molecule. We substitute the transition matrix element in Equation (15) to the expression for the absorption cross section in Equation (8) and obtain

(A4) $$\begin{array}{cc} \sigma (t_{p})= & \frac{4\pi^{2}}{\omega_{in}}\sum_{m,j}{\left|e^{ik\cdot R_{m,j}}\sum_{m^{\prime }}\Omega_{F}^{m^{\prime }}\left(t_{p}\right)d_{j}\delta_{m,m^{\prime }}\right|}^{2}\\ = & \frac{4\pi^{2}}{\omega_{in}}\sum_{j}d_{j}^{2}\sum_{m}{\left|\Omega_{F}^{m}\left(t_{p}\right)\right|}^{2}. \end{array}$$

We substitute the definition for $\Omega_{F}^{m}$ and obtain

(A5) $$\begin{array}{cc} \sigma (t_{p})= & \frac{\pi^{3}E_{0}^{2}\tau^{2}}{2\ln2\omega_{in}}e^{-\frac{{\left(\langle E_{F}\rangle -\langle E\rangle -\omega_{in}\right)}^{2}\tau^{2}}{4\ln2}}\sum_{j}d_{j}^{2}\sum_{n,n^{\prime }}e^{-i\left(E_{n}-E_{n^{\prime }}\right)t_{p}}\sum_{m}a_{nm}a_{n^{\prime }m}^{*}. \end{array}$$

Let us look into the sum $\sum_{m}a_{nm}a_{n^{\prime }m}^{*}$ in more details by revisiting the definition of $a_{nm}$ as $c_{n}^{\tilde{m}*}{\tilde{c}}_{n}^{m}$ and that $c_{n}^{m}$ are the coefficients of an orthonormal basis set.

(A6) $$\begin{array}{cc} \sum_{m}a_{nm}a_{n^{\prime }m}^{*} & =c_{n^{\prime }}^{\tilde{m}}c_{n}^{\tilde{m}*}\sum_{m}c_{n}^{m}c_{n^{\prime }}^{m*}=c_{n}^{\tilde{m}}c_{n}^{\tilde{m}*}\delta_{n,n^{\prime }}. \end{array}$$

Thus, the absorption cross section becomes

(A7) $$\begin{array}{cc} \sigma (t_{p})= & \frac{\pi^{3}E_{0}^{2}\tau^{2}}{2\ln2\omega_{in}}e^{-\frac{{\left(\langle E_{F}\rangle -\langle E\rangle -\omega_{in}\right)}^{2}\tau^{2}}{4\ln2}}\sum_{j}d_{j}^{2}\sum_{n,n^{\prime }}\delta_{n,n^{\prime }}{\left|c_{n}^{\tilde{m}}\right|}^{2}e^{-i\left(E_{n}-E_{n^{\prime }}\right)t_{p}}. \end{array}$$

We note that $\sum_{n}{\left|c_{n}^{\tilde{m}}\right|}^{2}=1$. This considerably simplifies the expression for the cross section, which becomes simply a time-independent function in Equation (16).

We now substitute the transition matrix element in Equation (17) and obtain the new expression for the absorption cross section

(A8) σ(tp)=4π2ωin∑m,jdjΩFm(tp)+d¯jΩFm±1(tp)2=4π2ωin∑m,jdj2|ΩFm(tp)|2+2djd¯jReΩFm(tp)ΩFm+1(tp)+ΩFm(tp)ΩFm−1(tp).

Here, we neglected the term proportional to ${\bar{d}}_{j}^{2}$ since it is much smaller than the other terms. From the analysis above, we already obtained that the first term proportional to $d_{j}^{2}$ provides a time-independent contribution. Substituting the definition of $\Omega_{F_{m,j,e}}$, we obtain that the time-dependent cross section is given by

(A9) σ(tp)=π3E02τ22ln2ωine−(EF−〈E〉−ωin)2τ24ln2×∑jdj2+2djd¯jRe∑m,n,n′an′m+1∗anm+an′m−1∗anme−i(En−En′)tp.

### Appendix A.4. Derivation of X-ray Absorption cross Section for Transitions above Fermi Level

The transition matrix elements in the case, then the core hole and the valence hole are located at the same molecule ($m=m^{\prime }=m^{\prime \prime }$) may deviate from $d_{j}^{\prime }$ due to correlation effects within the same molecule. However, this will not influence the final conclusion and we do not distinguish such transition matrix elements for simplicity.

The matrix elements $\langle \Phi_{F_{m,j,{\tilde{m}}^{\prime },m^{\prime \prime }}}|e^{ik\cdot r}\epsilon \cdot \hat{d}|\Phi_{m^{\prime }}\rangle$ are nonzero for $m^{\prime }={\tilde{m}}^{\prime }$ as discussed in Section 3.2. This means that a Kronecker delta $\delta_{m^{\prime },{\tilde{m}}^{\prime }}$ appears in the expression for the time-resolved cross section in Equation (8) and the summation over Frenkel basis states inside the square of absolute values reduces to one term. Substituting the expression for the transition matrix elements in Equation (21) to Equation (8), we obtain

(A10) σ(tp)=π3E02τ22ln2ωin∑m,j,m′,m″e−〈EF〉−〈E〉−ωin2τ24ln2δm,m″dj′+δm,m″±1d¯j′∑nanm′e−iEntp2=π3E02τ22ln2ωine−〈EF〉−〈E〉−ωin2τ24ln2∑m,j,m′,m″dj′2δm,m″∑nanm′e−iEntp2+2∑m,j,m′,m″δm,m″δm,m″±1dj′d¯j′Re∑nanm′e−iEntp∑nanm′∗eiEntp+∑m,j,m′,m″d¯j′2δm,m″±1∑nanm′e−iEntp2

The term proportional to $d_{j}^{\prime }{\bar{d}}_{j}^{\prime }$ becomes zero, since $\delta_{m,m^{\prime \prime }}\delta_{m,m^{\prime \prime }\pm 1}=0$ and we obtain

(A11) $$\begin{array}{cc} \sigma (t_{p})= & \frac{\pi^{3}E_{0}^{2}\tau^{2}}{2\ln2\omega_{in}}e^{-\frac{{\left(\langle E_{F}\rangle -\langle E\rangle -\omega_{in}\right)}^{2}\tau^{2}}{4\ln2}}\left(\sum_{m,j,m^{\prime \prime }}{d_{j}^{\prime }}^{2}\delta_{m,m^{\prime \prime }}+\sum_{m,j,m^{\prime \prime }}{\bar{d_{j}^{\prime }}}^{2}\delta_{m,m^{\prime \prime }\pm 1}\right)\sum_{m^{\prime }}{\left|\sum_{n}a_{nm^{\prime }}e^{-iE_{n}t_{p}}\right|}^{2} \end{array}$$

Applying Equation (A6), we obtain $\sum_{m^{\prime }}{\left|\sum_{n}a_{nm^{\prime }}e^{-iE_{n}t_{p}}\right|}^{2}=\sum_{n,n^{\prime }}{\left|{\tilde{c}}_{n}^{\tilde{m}}\right|}^{2}\delta_{n,n^{\prime }}=1$. The summation over $\sum_{m,j,m^{\prime \prime }}\delta_{m,m^{\prime \prime }}$ gives the number of the molecules in the chain and the summation $\sum_{m,j,m^{\prime \prime }}\left(\delta_{m,m^{\prime \prime }+1}+\delta_{m,m^{\prime \prime }-1}\right)$ gives twice the number of nearest-neighbour molecules.

It would be more precise to distinguish the case, then $m=m^{\prime }=m^{\prime \prime }$, i.e., the core excitation and the valence excitation are at the same molecular site in the final state

(A12) $$\begin{array}{c} \langle \Phi_{F_{m,j,m^{\prime },m^{\prime \prime }}}|e^{ik\cdot r}\epsilon \cdot \hat{d}|\Phi_{m^{\prime }}\rangle =e^{ik\cdot R_{m,j}}\left(\delta_{m,m^{\prime \prime }}d_{j}^{\prime }+\delta_{m,m^{\prime \prime }\pm 1}{\bar{d}}_{j}^{\prime }+\delta_{m,m^{\prime }}\delta_{m,m^{\prime \prime }}{\tilde{d}}_{j}^{\prime }\right). \end{array}$$

${\tilde{d}}_{j}^{\prime }$ would be a correction for electronic correlations at the same molecular site. However, this would not change the conclusion that the absorption cross section would remain time-independent and we neglect the correction for simplicity.
